# Overweight and Obesity: The Interplay of Eating Habits and Physical Activity

**DOI:** 10.3390/nu15132896

**Published:** 2023-06-27

**Authors:** George Moschonis, Gina Louise Trakman

**Affiliations:** Discipline of Dietetics and Human Nutrition, Department of Sport, Exercise and Nutrition Sciences, School of Allied Health, La Trobe University, Melbourne 3086, Australia

The prevalence of overweight and obesity has been steadily increasing over the last 50 years, with worldwide obesity rates tripling since 1975, thus reaching pandemic proportions. Overweight and obesity are predominately a result of a sustained positive energy balance, stemming from a combination of excess dietary energy intake (mainly due to poor eating habits) and reduced energy expenditure (due to lack of physical activity and prolonged sedentary activities). The worldwide COVID-19 pandemic and associated lockdowns influenced dietary intake and energy expenditure, with mixed impacts on weight status. Snacking frequency, lack of access to fresh produce, alcohol consumption, emotional eating, and sedentary behaviors (related to a decreased necessity for incidental exercise and inability to engage in organized exercise) have been noted as risk factors for weight gain during self-quarantine. In contrast, replacing takeaway and restaurant meals with home-cooked meals has been associated with weight loss. Overweight and obesity significantly increase cardiometabolic risk throughout life, while in adulthood they are also associated with a high risk of morbidity and mortality from non-communicable chronic diseases, primarily cardiovascular disease, type 2 diabetes mellitus, musculoskeletal disorders, and certain types of cancer. In addition to the consequences on physical health, obesity also has an important impact on mental health since individuals with excess body weight often experience stigmatization and social isolation, which is associated with chronic stress and depression. As such, the prevention and treatment of overweight and obesity need to become a public health priority and programs targeting eating habits and physical activity should be implemented in both children and adults at community and individual levels. The aim of this Special Issue was to bring together a selection of original research manuscripts to showcase the latest evidence on the role of eating habits, physical activity, and their interplay in the configuration of energy balance on obesity prevention and management.

Overweight and obesity affect more than one-third of the global population, with more than 1.9 billion adults and nearly 400 million children and adolescents being overweight or obese. The pandemic proportions of excess body weight were also confirmed by the studies published in this Special Issue. Moschonis et al. [1] presented overweight and obesity prevalence data from six European countries (i.e., Belgium, Bulgaria, Finland, Greece, Hungary, and Spain) as part of the Fee4Diabetes study and reported that nearly 25.5% of children and adolescents were overweight or obese. Much higher prevalence rates were reported for the adult participants also examined in this study (i.e., children’s parents) with the prevalence of overweight and obesity reaching 42.5% and 68.5% in adult females and males, respectively. Moschonis et al. [1] also showed that the prevalence of overweight and obesity in children and adolescents was significantly higher in southern European countries (i.e., Greece and Spain), where it was reaching 32%. This is in line with the prevalence of 35.2% and 38% reported by Aragon-Matin et al. [2] and Oliveira et al. [3], respectively, for school-aged Spanish children. Similar findings also apply to other parts of the world, with the study of Tan et al. [4] showing that the prevalence of overweight and obesity among young adults in Malaysia was 39.8% before the COVID-19 pandemic, increasing to 41.5% during the COVID-19 pandemic.

The COVID-19 pandemic has been recently added to the list of factors that seem to have a negative impact on dietary and physical activity-related behaviors, thus contributing to a gradual weight gain in the population. Regarding other factors reported in this Special Issue to be significantly associated with overweight and obesity and, consequently, to the underlying eating and physical activity-related behaviors, Moschonis et al. [1] observed that childhood obesity was positively and strongly associated with the obesity status of their parents, with one in two children in Europe being overweight when both parents were obese. This finding is indicative of the “obesogenic environment” that obese parents usually create and maintain both for themselves but also for their children. This “obesogenic environment” usually comprises lower engagement with physical activities, more screen and other sedentary behaviors, and higher consumption of calorie-dense foods, which can gradually lead to weight gain in all family members. Moschonis et al. [1] also reported that the prevalence of overweight and obesity among children in Europe was higher in children of older, unemployed, and less educated parents, with these associations being possibly mediated by a less healthy diet and lower levels of physical activity within these families. Children’s knowledge of eating, nutrition, and physical activity was also highlighted by Oliveira et al. [3] as another important factor that determines healthy eating habits and physical activity levels in children.

Excess energy intake contributes to positive energy intake and obesity prevalence. Tan et al. [4] employed a web-based, anonymous survey and a modified version of the dietary diversity questionnaire to assess sociodemographic data, height, pre-pandemic body weight (retrospectively recalled), and current (15 months post first COVID lockdown) body weight of young adults (18–30) living in Malaysia. Using a hierarchal multiple regression model they found an association between increased consumption of cereals and grains and fats and oils and weight gain, with a corresponding association between decreased consumption of these food groups and weight loss. These findings are unsurprising given that these food groups are known to have a high energy density. However, since the dietary questionnaire that was utilized focused solely on whether consumption of pre-specified food groups was altered, the contribution of fats and oil and cereals and grains to total energy intake could not be ascertained. Interestingly, in this study, there was also a correlation between the increased intake of food groups that contribute significantly to fat intake (i.e., milk and dairy products, nuts and seeds) and weight loss. This finding highlights the importance of considering dietary sources of fat when assessing the impact of total fat intake on weight status. Fats, especially unsaturated fats, consumed through unprocessed foods that are also micronutrient rich, are an important part of a balanced diet and likely do not need to be restricted to maintain a “healthy” body weight. In relation, the frequency of eating and meal timing may play a role in weight status, with Aragon-Matin et al. [2] reporting that consuming breakfast more regularly was positively associated with better body weight status in schoolchildren.

Physical activity, which includes incidental exercise associated with activities of daily living and planned exercise, impacts on energy balance. Exercise can also improve physical fitness and result in a host of benefits such as improved mood and decreased risk of chronic disease. Aragon-Matin et al. [2] found that following healthier lifestyles and having good physical fitness, measured by the international fitness scale, was associated with healthier body weight status in schoolchildren. Likewise, unhealthy lifestyle behaviors and poor physical fitness increased the risk of being overweight or obese ten-fold. Aragon-Matin et al. [2] deemed breakfast consumption, fruit and vegetables, participation in school physical education and extra-curricular sports activities, and adequate sleep as healthy lifestyle behaviors. In contrast, consuming foods outside the home, consuming processed baked goods, fried foods, snacks, sugary soft drinks, and screen time were considered unhealthy lifestyle behaviors. The study authors found a significant independent association between screen time and body weight, which was statistically significant in boys. Chen et al. [5] were also interested in the impact of screen time on weight status. In this context, they conducted a cross-sectional analysis of baseline data from the Physical Activity and Health in Older Women Study (PAHIWOS cohort study) to examine associations between sedentary time, short video viewing, and overweight/obesity in Chinese community-dwelling older women. Both sedentary time and short video-viewing time were associated with indicators of overweight and obesity, but only the associations with short video viewing remained significant in a fully adjusted model that corrected for moderate–vigorous-intensity physical activity. Of note, food videos had a greater effect on overweight/obesity than non-food videos. These results suggest that adequate moderate–vigorous physical activity may ameliorate the negative impacts of sedentary time and that the nature of the content being watched during screen time influences the relationship between screen time and body weight status. Accordingly, advice regarding screen time, sedentary time, and physical activity needs to be nuanced.

Findings from this Special Issue suggest that adjustment of macronutrient intake and dietary supplements may have adjunct roles in maximizing the benefits of physical activity. Tsujino et al. [6] conducted a placebo-controlled, double-blind, randomized cross-over trial to assess the impact of continuous ingestion of 2 g of medium-chain triglycerides (MCT) on substrate metabolism during low-intensity activity in Japanese adults (35–65) who had a BMI of 25–30 kg/m^2^. Consumption of the MCT supplement increased saturated fat intake and led to a subsequent increase in fat oxidation and decreased Respiratory Exchange Ration (RER) during 30 min of cycling on an ergometer. The authors surmised that while total energy expenditure during activity did not change, the potential for fat burning during incidental activities, which have a similar METS to the cycling that was undertaken, and increased post-prandial fat oxidation mean that MCT oil has possible anti-obesogenic effects.

In conclusion, the studies included in the Special Issue *Overweight and Obesity: The Interplay of Eating Habits and Physical Activity* showed that there is a constant interplay between eating habits and physical activity, which determine energy balance and consequently affect body weight status. Gaining a better understanding of the exact behaviors that configure energy balance in different life stages and populations and the personal, social, demographic, and environmental determinants of these behaviors provides a better basis for developing future, tailored interventions for tackling obesity and related chronic diseases. The evidence summarized in this Special Issue contributes towards the fulfilment of this objective.
